# Postoperative normocalcemic hyperparathyroidism: a comprehensive analysis of 510 cases

**DOI:** 10.55730/1300-0144.6210

**Published:** 2026-02-04

**Authors:** Alparslan ERTENLİCE, İbrahim KILINÇ, Mustafa ORUÇ

**Affiliations:** 1 Department of General Surgery, Faculty of Medicine, Ankara Yıldırım Beyazıt University, Ankara, Turkiye; 2 Department of General Surgery, Ankara Bilkent City Hospital, Ankara, Turkiye

**Keywords:** Normocalcemic hyperparathyroidism, primary hyperparathyroidism, parathyroidectomy, preoperative PTH, alkaline phosphatase, parathyroid hyperplasia

## Abstract

**Background/aim:**

Postoperative normocalcemic hyperparathyroidism (NC-HPT) is defined as elevated parathyroid hormone (PTH) levels despite normal calcium levels after parathyroidectomy for primary hyperparathyroidism (PHPT). This study investigates the demographic, biochemical, and histopathological factors associated with NC-HPT in a large patient cohort and presents the follow-up outcomes.

**Materials and methods:**

The initial study sample included 571 patients who underwent surgery for PHPT between January 2018 and January 2024, 510 of whom were analyzed retrospectively after the application of the exclusion criteria. Preoperative and postoperative biochemical parameters, bone mineral density, pathological findings, and follow-up data were evaluated. NC-HPT was defined as postoperative PTH elevation in the presence of normocalcemia. Logistic regression and receiver operating characteristic curve analyses were performed.

**Results:**

NC-HPT developed in 104 (20.4%) of the 510 patients analyzed in the study. The condition peaked in the third postoperative month and then gradually declined, with 86.5% of cases resolving by month 30. Univariate analysis revealed elevated preoperative PTH, high alkaline phosphatase (ALP), and parathyroid hyperplasia to be significant predictors. In multivariate analysis, only preoperative PTH level (OR: 1.003; 95% CI: 1.002–1.005; p < 0.001) and hyperplasia (OR: 3.311; 95% CI: 1.277–8.547) remained independent risk factors. ROC analysis identified a preoperative PTH cutoff value of 298 pg/mL with an AUC of 0.665 (95% CI: 0.608–0.723). No significant associations were observed with other demographic, biochemical, or radiological parameters.

**Conclusion:**

NC-HPT occurs in approximately 20% of patients undergoing surgery for PHPT but often resolves during follow-up. Elevated preoperative PTH levels and parathyroid hyperplasia are independent predictors of NC-HPT, and patients with these characteristics should be monitored more closely in the postoperative period.

## 1. Introduction

Primary hyperparathyroidism (PHPT) is the most common cause of hypercalcemia in outpatients [1]. Excessive secretion of parathyroid hormone (PTH) from the parathyroid glands leads to elevated serum calcium levels. Treatment success in PHPT is defined as the normalization of calcium levels after surgery and maintenance of this state for at least 6 months [2]. Calcium and PTH levels are both expected to decrease in PHPT following parathyroidectomy, and persistent or recurrent disease may be indicated if the levels remain elevated in postoperative follow-up. Calcium levels may remain within the normal range while PTH levels stay elevated in a reported 8–40% of cases—a condition known as postoperative normocalcemic hyperparathyroidism (NC-HPT) [3]. Several etiological factors and mechanisms have been proposed for NC-HPT; however, the clinical significance of elevated PTH levels remains unclear [4].

In the present study, a large cohort of 510 cases was assessed with the primary goal of identifying demographic, biochemical, and histopathological markers potentially associated with NC-HPT, thereby clarifying inconsistencies in the literature. As a secondary goal, we evaluated the follow-up outcomes of the study population.

## 2. Materials and methods

### 2.1. Study design and patients

Included in this retrospective observational study were 571 patients who underwent parathyroidectomy for PHPT at a tertiary care center between January 2018 and January 2024. Patients with persistent hypercalcemia (n = 6), chronic renal failure (n = 7), familial syndromes (n = 3), incomplete medical records, and/or missing follow-up data (n = 45) were excluded, and the study was subsequently concluded with a total of 510 patients.

PHPT diagnosis in all patients was established based on elevated serum calcium (>10.5 mg/dL) and elevated plasma parathyroid hormone PTH levels (>80.1 pg/mL). The localization of the parathyroid gland(s) responsible for PHPT was determined by cervical ultrasonography and sestamibi SPECT imaging. When deemed necessary, cervical computed tomography (CT) was performed.

The outcome of interest was defined as postoperative normocalcemic PTH elevation (referred to as NC-HPT in the present study), characterized by a PTH level of >80.1 pg/mL with concurrent normal serum calcium levels at any postoperative follow-up timepoint, regardless of duration.

### 2.2. Data collection

The patients’ demographic data, including age, sex, date of surgery, and date of the last evaluation, were documented. Laboratory data included preoperative serum calcium, parathyroid hormone (PTH), serum phosphorus, alkaline phosphatase (ALP), and vitamin D levels. Corrected calcium was calculated using the Payne formula (Corrected Ca [mg/dL] = [0.8 × (4.0 – albumin [g/dL])] + measured Ca). Serum 25-hydroxyvitamin D [25(OH)D] and PTH levels were measured using a chemiluminescent immunoassay on a Siemens Atellica IM analyzer, with reference ranges of 30–100 ng/mL and 15–65 pg/mL, respectively. All vitamin D measurements were standardized to ng/mL. Other biochemical parameters were analyzed using a Siemens Atellica CH analyzer. The institutional reference ranges were as follows: albumin 3.2–4.8 g/dL, ALP 42–98 U/L, calcium 8.7–10.4 mg/dL, phosphorus 2.4–5.1 mg/dL, magnesium 1.3–2.7 mg/dL, BUN 19–49 mg/dL, creatinine 0.5–1.1 mg/dL, uric acid 3.1–7.8 mg/dL, urinary calcium 100–300 mg/24 h, urinary phosphorus 0.4–1.3 g/24 h, and eGFR >90 mL/min/1.73 m^2^. For preoperative values, measurements obtained in the week prior to surgery were considered. Postoperative PTH levels were recorded on postoperative day 1 and at months 1, 3, 6, 12, 24, and 30.

Bone health was evaluated based on bone mineral density (BMD) and T-scores obtained from dual-energy X-ray absorptiometry (DXA) of the lumbar spine (L1–L4). Measurements were taken using a GE/Lunar Prodigy densitometer (GE Healthcare, Madison, WI, USA). Instead of applying a binary classification, BMD (g/cm^2^) and T-score values were analyzed as continuous variables for the assessment of bone status.

The maximum lesion diameters (mm) and volumes (mm^3^) were recorded based on preoperative ultrasonography reports. Parathyroid tumors were considered ellipsoid objects, and tumor volume was calculated using the formula 4/3 πabc or 4/3 πab^2^ (where a, b, and c represent the radii).

### 2.3. Surgical technique and follow-up

Focused parathyroidectomy was performed in patients with a solitary adenoma. In cases where multiple gland involvement confined to one side was noted on preoperative imaging, a unilateral exploration was carried out. Bilateral neck explorations were performed when the adenoma could not be localized preoperatively, when preoperative findings suggested bilateral multiglandular disease, or when intraoperative PTH measurements and frozen section analyses failed to confirm adequate parathyroidectomy. All patients underwent intraoperative PTH monitoring to confirm the complete excision of the hyperfunctioning tissue, as part of the standard surgical protocol applied in our clinic. A ≥50% decline in intraoperative PTH compared with the baseline value and/or a decrease into the predefined normal range (<80.1 pg/mL) was considered indicative of successful parathyroidectomy. In some patients, the complete intraoperative excision of the abnormal glands was confirmed by pathological examination.

Calcium and PTH levels were measured at regular intervals within the first 6 months following surgery, and the period was extended when abnormalities in these parameters were detected (mean duration = 2.5–3 years). Ensuring immediate postoperative normocalcemia was considered sufficient for defining the study cohort—allowing for the assessment of PTH dynamics starting from the first postoperative day while ensuring the exclusion of persistent hypercalcemia.

### 2.4. Statistical analysis

Data were analyzed using IBM SPSS Statistics (version 26.0, IBM Corp., Armonk, NY, USA). The distribution of continuous variables was assessed using a Shapiro–Wilk test and by examining skewness and kurtosis. Normally distributed variables were expressed as mean ± standard deviation, and nonnormally distributed variables as medians (interquartile range). Categorical variables were presented as n (%). Group comparisons of continuous variables were made using Student’s t-test or the Mann–Whitney U test, depending on the distribution. Categorical variables were compared with a chi-square or Fisher’s exact test. A multivariate logistic regression model was constructed using the “Enter” method to identify independent predictors of NC-HPT. The model was adjusted for the clinically relevant covariates established in the literature—including age, sex, eGFR, vitamin D, calcium, and phosphorus—regardless of their univariate significance, to control for potential confounding. The linearity assumption for continuous variables (e.g., PTH) was checked using the Box-Tidwell procedure. Model fitness was evaluated using the Hosmer–Lemeshow goodness-of-fit test, and explanatory power was assessed with Nagelkerke R^2^. The results were reported as odds ratios (OR) with 95% confidence intervals (CI). For significant continuous variables, receiver operating characteristic (ROC) analysis was performed to calculate the area under the curve (AUC), 95% CI, sensitivity, specificity, positive predictive value, and negative predictive value. The identified cutoff values were further tested in regression models. A p-value of <0.05 was considered significant. Calculations for point prevalence at specific time points were based on the complete cohort (n = 510), as patients with missing follow-up data had been excluded from the study design.

## 3. Results

**Figure 1:**
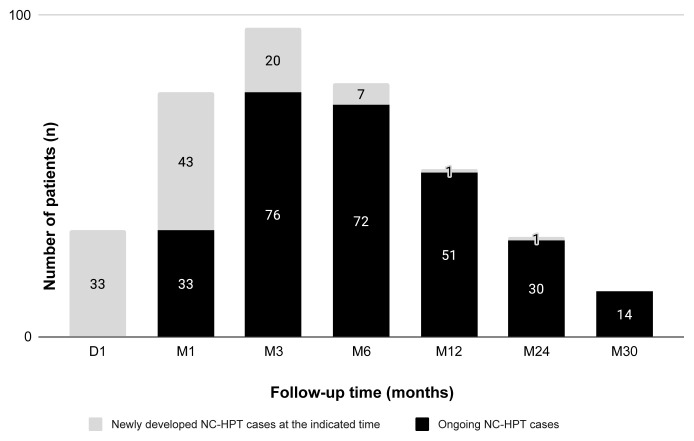
Ongoing and newly developed NC-HPT cases during follow-up. **Note:** The number of cases presented at each time point corresponds to the entire study cohort (n = 510). The denominator for the calculation of prevalence remained constant across all follow-up intervals.

**Figure 2:**
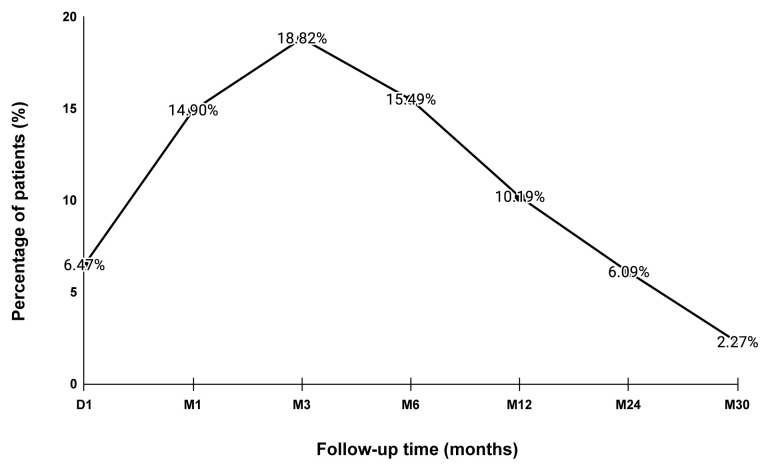
Point prevalence of postoperative normocalcemic PTH elevation over time. Note: Percentages at each time point are calculated based on the total study cohort (n = 510).

Of the 510 patients included in the study, 104 (20.4%) developed NC-HPT and were followed up for a mean duration of 29.5 ± 3.0 months. In the remaining 406 patients (79.6%), calcium and PTH levels both normalized within the postoperative period. The number of patients with NC-HPT at the postoperative day 1 and months 1, 3, 6, 12, 24, and 30 timepoints was 33, 76, 96, 79, 52, 31, and 14, respectively. The 33 cases identified on postoperative day 1 were followed by 43 new cases at month 1, 20 at month 3, seven at month 6, one at month 12, and one at month 24 (Figure 1). The cumulative incidence of NC-HPT was 20.39%. The point prevalence rates were 6.47% on postoperative day 1, and 14.9%, 18.82%, 15.49%, 10.19%, 6.07%, and 2.27% at months 1, 3, 6, 12, 24, and 30, respectively (Figure 2).

### 3.1. Demographic, clinical, and biochemical characteristics

There was no significant difference in the mean ages of the patients who developed NC-HPT and those who did not (52.9 ± 12.4 vs 52.7 ± 12.5 years; p = 0.466). The female-to-male ratio was also comparable (78.8% vs 79.3%; p = 0.917).

Preoperative calcium, phosphorus, 25(OH)D levels, BMD, and T-score did not differ significantly between the groups. In contrast, patients who developed NC-HPT had significantly higher preoperative PTH levels (252.5 [190–413.5] vs 193 [139–269] pg/mL; p < 0.001) and preoperative alkaline phosphatase (ALP) levels (118.5 [92.5–164] vs 111 [85–141] U/L; p = 0.003).

Postoperative corrected calcium levels were similar between the groups (8.75 ± 0.89 vs 8.73 ± 0.76 mg/dL; p = 0.866). Likewise, no significant association was found between NC-HPT development and preoperative or postoperative vitamin D supplementation, or postoperative calcium supplementation (Table 1).

### 3.2. Pathological findings

Pathological evaluation revealed adenoma to be the most common lesion (91.2%), although the proportion of hyperplasia was significantly higher in the NC-HPT group than in the non-NC-HPT group (8.7% vs 2.7%, p = 0.001), and the rate of parathyroid carcinoma was also higher in the patients who developed NC-HPT (2.9% vs 0.2%) (Table 2).

### 3.3. Univariate and multivariate analyses

In the univariate logistic regression analysis, higher preoperative PTH (OR: 1.002; 95% CI: 1.001–1.002; p < 0.001), elevated ALP (OR: 1.001; 95% CI: 1.000–1.002; p =0.035), and parathyroid hyperplasia (OR: 3.402; 95% CI: 1.371–8.443; p = 0.008) were identified as significant predictors of NC-HPT (Table 3).

In the multivariate logistic regression analysis, which was adjusted for age, sex, eGFR, vitamin D, calcium, and phosphorus, preoperative PTH (OR: 1.003, 95% CI: 1.002–1.005; p < 0.001) and histopathology (OR: 3.311, 95% CI: 1.277–8.547; p = 0.014) were identified as independent predictors of NC-HPT. Other variables included in the model did not show statistical significance (Table 4). The final model demonstrated good calibration (Hosmer–Lemeshow test p = 0.950) and discrimination (Nagelkerke R^2^ = 0.119). Parathyroid carcinoma, while significantly higher in the NC-HPT group in univariate analysis (p = 0.001), was excluded from the multivariate model due to the small number of cases (n = 4) to avoid sparse data bias. In a sensitivity analysis excluding these carcinoma cases, the primary results of the logistic regression remained unchanged.

### 3.4. ROC curve analysis

**Figure 3:**
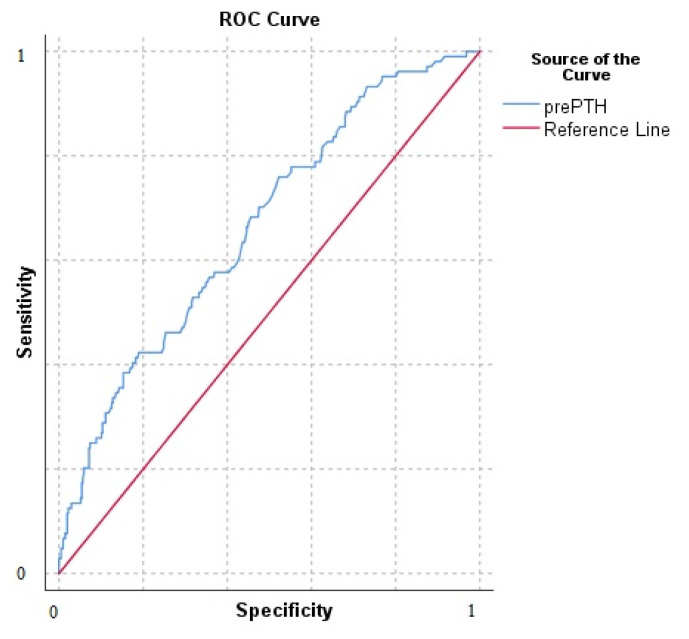
ROC curve of preoperative PTH level for predicting postoperative NC-HPT.

In the ROC curve analysis performed for preoperative PTH (Figure 3), the AUC was 0.665 (95% CI: 0.608–0.723; p < 0.001), indicating moderate discriminatory ability. When a cutoff value of 298 pg/mL was set, the sensitivity was 42.3%, specificity was 81.0%, the positive predictive value was 36.4%, and the negative predictive value was 84.6% (Table 5).

## 4. Discussion

The incidence of postoperative NC-HPT in patients operated on for PHPT has been reported to range between 8% and 40% [3]. Since NC-HPT may develop at different time points during the postoperative period, it is necessary to specify the time of assessment when reporting the incidence. In the present study, NC-HPT developed in 104 of the 510 patients who underwent surgery for primary hyperparathyroidism, and the cumulative incidence was calculated as 20.39%, which is consistent with earlier studies. In our study, the number of patients who developed NC-HPT increased from postoperative day 1 to a peak at month 3, and then progressively declined, reaching its minimum level at month 30. It can thus be inferred that the likelihood of NC-HPT increases in patients undergoing parathyroidectomy until the third month and gradually decreases thereafter. Previous studies have reported that NC-HPT may persist up to the 4th postoperative year [5]; however, in our cohort PTH levels had normalized by month 30 in 86.53% of patients. Benet-Muñoz et al. also reported a normalization rate of 90% at month 30 [6]. There remains a lack of consensus on the clinical significance of NC-HPT. Several authors have suggested that this condition is transient and clinically insignificant [5,7], while others claim that NC-HPT is not merely a temporary phenomenon, but rather a precursor of classical PHPT [3,8–11]. In our cohort, during a mean follow-up period exceeding 2 years, none of the patients—including those with histopathological evidence of hyperplasia—developed classical hyperparathyroidism.

The pathogenesis of postoperative NC-HPT has yet to be fully elucidated, and numerous preoperative and postoperative factors have been investigated related to the condition. In the present study, no significant association was found between NC-HPT and either age or sex, concurring with several reports suggesting that demographic factors are not determinative [2,12]. It has been reported that downregulation of PTH receptors in target organs, resulting from chronic exposure to elevated PTH levels, may play an important role in the pathogenesis of NC-HPT [13]. In the present study, elevated preoperative PTH was identified as an independent predictor of NC-HPT development. Although the OR for preoperative PTH appears to be numerically small (1.003 per pg/mL), this translates to a clinically significant risk increase over larger intervals (e.g., an approximately 16% increase in risk for every 50 pg/mL elevation in PTH levels). Furthermore, ROC curve analysis determined a cutoff value of 298 pg/mL for preoperative PTH—a threshold that demonstrates high specificity (81%) but relatively low sensitivity (42.3%). These findings suggest that elevated preoperative PTH levels may serve as a meaningful predictor of postoperative normocalcemic PTH elevation. However, given the limited sensitivity, this cutoff should not be interpreted as a definitive screening threshold for ruling out the disease. Rather, its main clinical utility lies in its high specificity—while values below 298 pg/mL do not guarantee safety, values exceeding this limit serve as a specific “warning sign” necessitating closer surveillance. To the best of our knowledge, the cutoff value we established for preoperative PTH is the first to be reported in the literature. Caldwell et al. [14] reported high preoperative PTH to be the strongest predictor of postoperative NC-HPT, while Benet-Muñoz et al. [6] and Duke et al. [15] identified preoperative PTH as an independent risk factor.

Preoperative ALP levels were also found to be associated with NC-HPT development but were not identified as an independent predictor in multivariate analysis (p = 0.224). This finding is consistent with previously reported data. In particular, Cao et al. [12] observed that preoperative ALP levels were significantly higher in patients with elevated postoperative PTH and interpreted this finding as an indicator of increased bone turnover. Likewise, De la Plaza Llamas et al. [2] suggested that while elevated ALP may represent a secondary contributing mechanism, it lacks independent predictive value. ALP is an indirect marker of bone turnover, and interacts strongly with primary biochemical parameters such as PTH and vitamin D levels. It therefore appears biologically plausible that ALP achieved statistical significance in the univariate analysis but lost its independent effect in the multivariate model. After adjusting for other covariates, although elevated ALP may be considered an auxiliary marker for the development of NC-HPT, it is not a strong predictor on its own.

In the present study, no significant association was found between preoperative calcium or phosphorus levels and the development of postoperative NC-HPT. This finding is consistent with the majority of studies reported in the literature [6,14,16–18]. On the other hand, some studies [3,12,19] have reported a statistical association between elevated preoperative calcium levels or reduced phosphorus levels and postoperative PTH elevation. In several of these studies, however, the association was observed only in univariate analysis and did not persist in multivariate models. For example, in the study by Kota et al. [3], lower phosphorus levels were significant in univariate analysis but not in multivariate analysis. It should also be noted that the number of cases in the studies reporting an association between calcium or phosphorus and NC-HPT was smaller than that in our cohort. Overall, these findings suggest that preoperative calcium and phosphorus levels are not independent determinants of NC-HPT development.

Impaired renal function has also been implicated in the pathogenesis of postoperative PTH elevation. It is well known that renal dysfunction affects parathyroid function, and that prolonged hypercalcemia can reduce glomerular filtration [20]. However, similar to the findings of Benet-Muñoz et al. [6], no significant difference in eGFR values was observed between the groups in the present study.

There have been studies reporting that vitamin D deficiency contributes to PTH elevation after parathyroidectomy [7,21,22], although the role of vitamin D supplementation in the development of NC-HPT remains controversial [23,24]. Similar to the findings of Benet-Muñoz et al. [6], no significant association between vitamin D levels or vitamin D supplementation and NC-HPT development was observed in the present study. As approximately 50% of the global population lives with vitamin D deficiency [25], our findings suggest that vitamin D levels play only a minor role in the pathophysiology of NC-HPT.

Previous studies have suggested that postoperative NC-HPT may develop as a result of mild hungry bone syndrome [2,4,12,26]; however, the validity of this claim has been questioned by other authors [13]. In the present study, no statistically significant differences were observed between the patients who developed NC-HPT and those who did not with respect to the parameters associated with hungry bone syndrome (e.g. postoperative calcium levels, postoperative calcium supplementation, BMD, and T-scores). Therefore, we also view with skepticism the hypothesis that a mild hungry bone syndrome plays a role in the pathophysiology of NC-HPT. Beyond these factors, several earlier studies have reported an association between larger adenoma size and NC-HPT [12,17], although this has not been confirmed in larger cohorts [14]. In our study, no significant association was observed between lesion diameter or volume and NC-HPT development.

The most notable histopathological finding in patients who developed NC-HPT in the present study was the presence of hyperplasia, which remained a significant predictor in the multivariate analysis. In contrast, no association was found between NC-HPT development and pathologies such as adenoma, atypical parathyroid tumor, or carcinoma. Although parathyroid carcinoma was more frequent in the NC-HPT group, it was excluded from the multivariate model due to the limited number of cases. This finding is consistent with previously published data. Several earlier studies have also reported that hyperplasia may predispose patients to postoperative PTH elevation [2,14,19]. From a pathophysiological perspective, this may be explained by the higher cellular burden in hyperplastic glands, which could result in delayed normalization or persistently elevated PTH levels after surgery. It can thus be suggested that patients with histopathological evidence of hyperplasia should be monitored closely during postoperative follow-up.

## 5. Conclusion

NC-HPT is a common condition occurring in approximately 20% of patients undergoing surgery for PHPT. Our findings support the pathophysiological hypothesis that normocalcemic PTH elevation may be related to target-organ receptor downregulation secondary to high preoperative PTH levels. Elevated preoperative PTH and the presence of hyperplasia were identified as independent predictive factors in our study. Notably, the NC-HPT had resolved by the 30th postoperative month in 86.5% of the patients.

The strengths of this study include its large sample size and its inclusion of detailed biochemical and parametric analyses. In contrast, its retrospective design and the relatively limited follow-up duration can be acknowledged as limitations.

## Figures and Tables

**Table 1:** Univariate associations between factors and the development of NC-HPT.

	Overall n = 510	NC-HPT (+) n = 104 (20.4%)	NC-HPT (−) 406 (79.6%)	p
**Age (years)**	52.9 ± 12.4	53.7 ± 11.8	52.7 ± 12.5	0.466
**Sex Female**	404 (79.2%)	82 (78.8%)	322 (79.3%)	0.917
**Male**	106 (20.8%)	22 (21.2%)	84(20.7%)	
**Preoperative PTH (pg/mL)**	203 (147–294)	252.5 (190–413.5)	193 (139–269)	<0.001
**Preoperative corrected Ca (mg/dL)**	11.44 (11.04–11.87)	11.38 (11.08–11.99)	11.45 (11.04–11.85)	0.917
**Preoperative phosphorus (mg/dL)**	2.64 ± 0.57	2.59 ± 0.63	2.65 ± 0.55	0.292
**Preoperative ALP (U/L)**	112.5 (87–144)	118.5 (92.5–164)	111(85–141)	0.003
**Preoperative 25(OH)D (ng/mL)**	14.7 (10.4–21)	14.8 (10.8–20.4)	14.5(10.2–21.1)	0.927
**Postoperative corrected Ca (mg/dL)**	8.74 ± 0.79	8.75 ± 0.89	8.73 ± 0.76	0.866
**Lesion maximum diameter (mm)**	12.35 (9.1–17.5)	12.3 (9.1–18.4)	12.4 (9.1–17.3)	0.642
**Lesion volume (mm** **^3^** **)**	427.3 (167–1139.8)	416.7 (172.9–1387.1)	427.8 (166.2–1123.3)	0.663
**BMD (g/cm** **^2^** **)**	1.004 ± 0.177	0.997 ± 0.185	1.005 ± 0.175	0.652
**T-score**	−1.4 (−2.4 to −0.5)	−1.4 (−2.4 to −0.4)	−1.4 (−2.4 to −0.5)	0.923
**Preoperative Vit D intake**	228 (44.7%)	47 (45.2%)	181 (44.6%)	0.911
**Postoperative Vit D intake**	372 (72.9%)	79 (76%)	293 (72.2%)	0.437
**Postoperative Ca intake**	249(48.8%)	46(44.2%)	203 (50%)	0.294
**eGFR**	95.5 ± 18.9	92.7 ± 20.7	96.2 ± 18.4	0.089
**Mean follow-up duration (months)**	-	29.5 ± 3	6 (6–12)^a^	-

**Abbreviations:** ALP: alkaline phosphatase, BMD: bone mineral density, Ca: calcium, PTH: parathyroid hormone, 25(OH)D: 25-hydroxyvitamin D, Vit D: vitamin D, eGFR: estimated Glomerular Filtration Rate.

a Follow-up for the control group was concluded between 6 and 12 months upon confirmation of sustained normocalcemia, in accordance with the study protocol’s minimum monitoring requirement.

**Table 2:** Univariate associations between pathology and the development of NC-HPT.

Parathyroid pathology	Overall n = 510	NC-HPT (+) n = 104 (20.4%)	NC-HPT (−) 406 (79.6%)	p
Carcinoma	4 (0.8%)	3 (2.9%)	1 (0.2%)	0.001
Hyperplasia	20 (3.9%)	9 (8.7%)	11 (2.7%)	
Atypical parathyroid tumor	21 (4.1%)	5 (4.8%)	16 (3.9%)	
Adenoma	465 (91.2%)	87 (83.7%)	378 (93.1%)	

**Table 3:** Univariate logistic regression analysis for predictors of NC-HPT.

Variables	p	OR (CI 95%)
Preoperative ALP (U/L)	0.035	1.001(1–1.002)
Preoperative PTH (pg/mL)	<0.001	1.002(1.001–1.002)
Hyperplasia	0.008	3.402 (1.371–8.443)

**Abbreviations:** ALP: alkaline phosphatase, PTH: parathyroid.

**Table 4:** Multivariate logistic regression analysis of factors associated with NC-HPT.

Variables	p-value	OR (95% CI)
Preoperative PTH (pg/mL)	<0.001	1.003 (1.002–1.005)
Hyperplasia	0.014	3.311 (1.277–8.547)
Preoperative ALP (U/L)	0.224	0.999 (0.996–1.001)
eGFR (mL/min/1.73m^2^)	0.079	0.985 (0.969–1.002)
Preoperative calcium (mg/dL)	0.101	0.735 (0.508–1.062)
Preoperative phosphorus (mg/dL)	0.472	1.179 (0.753–1.847)
Age (years)	0.548	0.992 (0.967–1.018)
Preoperative 25(OH)D (ng/mL)	0.811	0.997 (0.969–1.025)
Sex (male vs female)	0.965	0.987 (0.555–1.755)

**Notes:** CI: Confidence interval, OR: Odds ratio. Model performance: The model demonstrated good fit (Hosmer–Lemeshow test p = 0.950) and discrimination (Nagelkerke R^2^ = 0.119).

* Histopathology variable evaluates the presence of hyperplasia compared to the reference group (Adenoma).

**Table 5:** ROC analysis of preoperative PTH level for predicting postoperative NC-HPT.

Variable	Cutoff (pg/mL)	Sensitivity (%)	Specificity (%)	PPV (%)	NPV (%)	AUC (95% CI)	p-value	LR+	LR−
Preoperative PTH (pg/mL)	298	42.3	81.0	36.4	84.6	0.665 (0.608–0.723)	<0.001	2.23	0.71

**Abbreviations:** PPV: Positive predictive value; NPV: Negative predictive value; LR+: Positive likelihood ratio; LR−: Negative likelihood ratio; PTH: Parathyroid hormone, AUC: Area under the curve; CI: Confidence interval.
